# Prevention of Congenital Malformations, &c.

**Published:** 1881-06

**Authors:** 


THE PREVENTION OF CONGENITAL MALFORMATIONS, DEFECTS AND DISEASES BY THE MEDICINAL AND NUTRITIONAL TREATMENT OF THE MOTHER DURING PREGNANCY. By J. C. Burnett, M.D., London. Editor *Homœopathic World.* Edited, with notes from Grauvogl, by T. C. Duncan, M.D., author of "Diseases of Infants," etc., pp. 26. Chicago, Duncan Brothers, 1881.

Dr. J. C. Burnett delivered an essay before the British Homœopathic Congress, on the above mentioned topics. The field embraced in this essay is as yet almost unexplored, is very important and exceedingly interesting. We can not agree with Dr. Burnett in his views as to the so-called "tissue remedies." We believe *any* homœopathic remedy which removes the morbid condition present, and thus allows of *proper* and healthy nutritive processes, will act the part of a "*tissue remedy.*" These conditions (malformations, etc.,) do not result from an absence of proper nutritive principles but from a *lack of proper assimilation.* Had not these mothers any symptoms during pregnancy, for which the simillimum remedy could be found? If the *calcarea sulph.* was prescribed for *symptoms* present in the pregnant mother and not for a *presumed* condition of the fœtus, we could agree with our author. Nevertheless, the essay is well worth a careful perusal, and the subject, of earnest study.

